# Cardiac Exposure in the Dynamic Conformal Arc Therapy, Intensity-Modulated Radiotherapy and Volumetric Modulated Arc Therapy of Lung Cancer

**DOI:** 10.1371/journal.pone.0144211

**Published:** 2015-12-02

**Authors:** Xin Ming, Yuanming Feng, Huan Liu, Ying Zhang, Li Zhou, Jun Deng

**Affiliations:** 1 Department of Biomedical Engineering, Tianjin University, Tianjin, China; 2 Department of Therapeutic Radiology, Yale University, New Haven, CT, United States of America; 3 Department of Therapeutic Radiology, Yale-New Haven Hospital, New Haven, CT, United States of America; 4 Center for Radiation Physics and Technology, West China Hospital Cancer Center, Sichuan University, Chengdu, China; University of California Davis, UNITED STATES

## Abstract

**Purpose:**

To retrospectively evaluate the cardiac exposure in three cohorts of lung cancer patients treated with dynamic conformal arc therapy (DCAT), intensity-modulated radiotherapy (IMRT), or volumetric modulated arc therapy (VMAT) at our institution in the past seven years.

**Methods and Materials:**

A total of 140 lung cancer patients were included in this institutional review board approved study: 25 treated with DCAT, 70 with IMRT and 45 with VMAT. All plans were generated in a same commercial treatment planning system and have been clinically accepted and delivered. The dose distribution to the heart and the effects of tumor laterality, the irradiated heart volume and the beam-to-heart distance on the cardiac exposure were investigated.

**Results:**

The mean dose to the heart among all 140 plans was 4.5 Gy. Specifically, the heart received on average 2.3, 5.2 and 4.6 Gy in the DCAT, IMRT and VMAT plans, respectively. The mean heart doses for the left and right lung tumors were 4.1 and 4.8 Gy, respectively. No patients died with evidence of cardiac disease. Three patients (2%) with preexisting cardiac condition developed cardiac disease after treatment. Furthermore, the cardiac exposure was found to increase linearly with the irradiated heart volume while decreasing exponentially with the beam-to-heart distance.

**Conclusions:**

Compared to old technologies for lung cancer treatment, modern radiotherapy treatment modalities demonstrated better heart sparing. But the heart dose in lung cancer radiotherapy is still higher than that in the radiotherapy of breast cancer and Hodgkin’s disease where cardiac complications have been extensively studied. With strong correlations of mean heart dose with beam-to-heart distance and irradiated heart volume, cautions should be exercised to avoid long-term cardiac toxicity in the lung cancer patients undergoing radiotherapy.

## Introduction

Radiation therapy combined with chemotherapy is usually the treatment of choice for small cell lung cancer (SCLC) and early stage non-small cell lung cancer (NSCLC) [1, 2]. Modern radiotherapy of lung cancer has seen the transition from 3D conformal radiation therapy (3D-CRT) to the more advanced and complicated techniques such as dynamic conformal arc therapy (DCAT), intensity-modulated radiotherapy (IMRT), and volumetric modulated arc therapy (VMAT) [3–6]. While tumor control has been improved with these techniques over the years, the radiation toxicity to the critical structures in the thoracic region remains to be a great concern.

Cardiac toxicity has been reported in the radiotherapy of breast cancer [7–18], mediastinal lymphoma [19–21], pediatric cancer [22, 23], esophageal cancer [24–26] and peptic ulcer disease [27, 28] for many years. Due to its proximity to the lesions, a fraction of heart volume usually received a relatively high dose, which largely contributed to radiation-induced heart diseases (RIHD) such as coronary artery disease, pericarditis, cardiomyopathy and valvular disease [29–35]. Some recent studies on the incidence rate of heart disease in women treated with radiotherapy for breast cancer indicated that the mean heart dose (MHD) were 0.4–14 Gy in breast cancer [11–16], 30 Gy in esophageal cancer [26] and 1.6–3.9 Gy in peptic ulcer disease [28]. The risk of cardiovascular diseases was found to be linearly correlated with MHD, and increased by 7.4% for each increase of 1 Gy in MHD for breast cancer [13], 10.2% in peptic ulcer [27], and 60% in pediatric cancer [23]. In a study of risk factors for pericardial effusion (PCE) in inoperable esophageal cancer patients treated with chemoradiation therapy, Wei *et al*. showed that high-dose radiation to the pericardium may strongly increase the risk of PCE and such a risk could be reduced by minimizing the dose-volume of the irradiated pericardium and heart [25]. Earlier investigations also suggested that the irradiated heart volume and the distance between the heart and the field edge can be used to predict the dose to the heart in the radiotherapy of breast cancer [17, 18, 36].

However, the data regarding cardiac exposure in the radiotherapy of lung cancer has largely been lacking [26, 37–41], especially for the techniques used in modern radiotherapy. In the radiation therapy oncology group (RTOG) 0623 for SCLC and RTOG 0617, 0813 and 0915 for NSCLC [42–45], the dose-volume limit was recommended as 60 Gy to <1/3, 45 Gy to <2/3, and 40 Gy to <100% of the heart, and the maximum point dose limit for the heart was generally suggested not to exceed 105% of the prescribed dose. With large prescription doses (45 to 60 Gy for SCLC; ≥66 Gy for NSCLC), the radiation dose to a fraction of heart volume incidentally irradiated by the treatment beams could be significant, depending on the location of the tumor and its involvement of the mediastinal lymph nodes [29–35]. Therefore, it is important and clinically significant to investigate the cardiac exposure for a better control of cardiac toxicity.

While it has been shown that modern approaches with better targeting and higher beam energy have contributed to reducing cardiac risks in the radiotherapy of breast cancer [10], a systematic investigation on the modern techniques such as DCAT, IMRT and VMAT for lung cancer treatment has been missing. Therefore, it would be clinically valuable and scientifically important to evaluate the radiation doses to the heart and other organ-at-risks in the modern radiotherapeutic management of lung cancer.

## Materials and Methods

### Patient Characteristics

140 lung cancer patients treated at the Department of Therapeutic Radiology of Yale-New Haven Hospital (YNHH) between October 1, 2007 and February 28, 2014 were included in this retrospective study, among which 25 were treated with DCAT, 70 with IMRT and 45 with VMAT. The average age of patients at diagnosis was 73 (range, 38–93), with 75 males and 65 females. Tumor locations were found evenly distributed among right upper lobe (RUL, 31), right middle lobe (RML, 26), right lower lobe (RLL, 30), left upper lobe (LUL, 29) and left lower lobe (LLL, 24). The average planning target volume (PTV) was 176.9 (range, 10.1–1266.8) cm^3^.

In this study, only patient's CTs, organ contours, age, gender and treatment plans were used and exported from Varian Eclipse treatment planning system. No patient's name or medical ID was recorded or used. For this research work, the patient data (CTs and contours) were analyzed anonymously. We submitted our IRB application to Yale University Human Investigation Committee on April 16, 2014 and received IRB approval on May 2, 2014. By the time when this research work was initiated in July 2014, the 140 patients have received their radiotherapy treatments and left YNHH. Since every patient treated at Department of Therapeutic Radiology of YNHH has signed their consent forms to receive radiotherapy treatments including CT scanning and the consent forms have been saved as part of electronic medical record, we therefore didn't seek to contact patients for another consent.

### Treatment planning

All patients were immobilized with both arms down in a CIVCO Body Pro-Lok immobilization device during simulation and treatment delivery (CIVCO Medical Solutions, Coralville, Iowa). For each patient, a 4DCT scan was acquired with Varian real-time position management system (RPM) v1.7.5 (Varian Medical Systems, Palo Alto, CA) on a 16-slice GE LightSpeed CT scanner with 2.5 mm slice thickness. The internal target volume (ITV) was contoured by the physicians based on the maximum intensity projection (MIP) of 4DCT. PTV was generated by adding a uniform margin of 7 mm to the ITV. All the critical structures including the heart, the bilateral lungs, the spinal cord, the trachea, the esophagus and the chest wall were also contoured. Treatment plans were generated on a Varian Eclipse treatment planning system v10.0.28 using 6 or 10 MV photon beams. Dose calculation was performed on the average intensity projection CT (reconstructed from the 4DCT) using the Anisotropic Analytical Algorithm (AAA) v10.0.28 with tissue heterogeneity correction turned on.

Specifically, all the DCAT plans were designed to deliver a total dose of 22.4 to 60 Gy to the PTV with 3 to 12 coplanar beams of 6 MV photon beams (except for one with 10 MV). For IMRT plans, a total of 4 to 12 non-coplanar, non-opposing beams of either 6 or 10 MV photons were used to deliver 30 to 70 Gy to the PTV. Although dose volume constraints for the target and organs-at-risk (OARs) may differ for individual IMRT plans, the following objectives were generally met: V20 < 30% for the bilateral lungs, the mean lung dose (MLD) < 20 Gy, and the maximum spinal cord dose < 45 Gy. For VMAT plans, a range of 10 to 70 Gy total doses was delivered with either 6 or 10 MV photons with the isocenter placed at the geometric center of PTV volume. Two partial arcs of 180° to 220° rotating in opposite directions were applied while avoiding as much of the OARs as possible. The dose volume constraints used in VMAT inverse planning were similar to those in IMRT plans. All 140 plans were normalized such that 100% isodose lines encompassed 95% of PTV.

### Cardiac exposure

**Fig 1:**
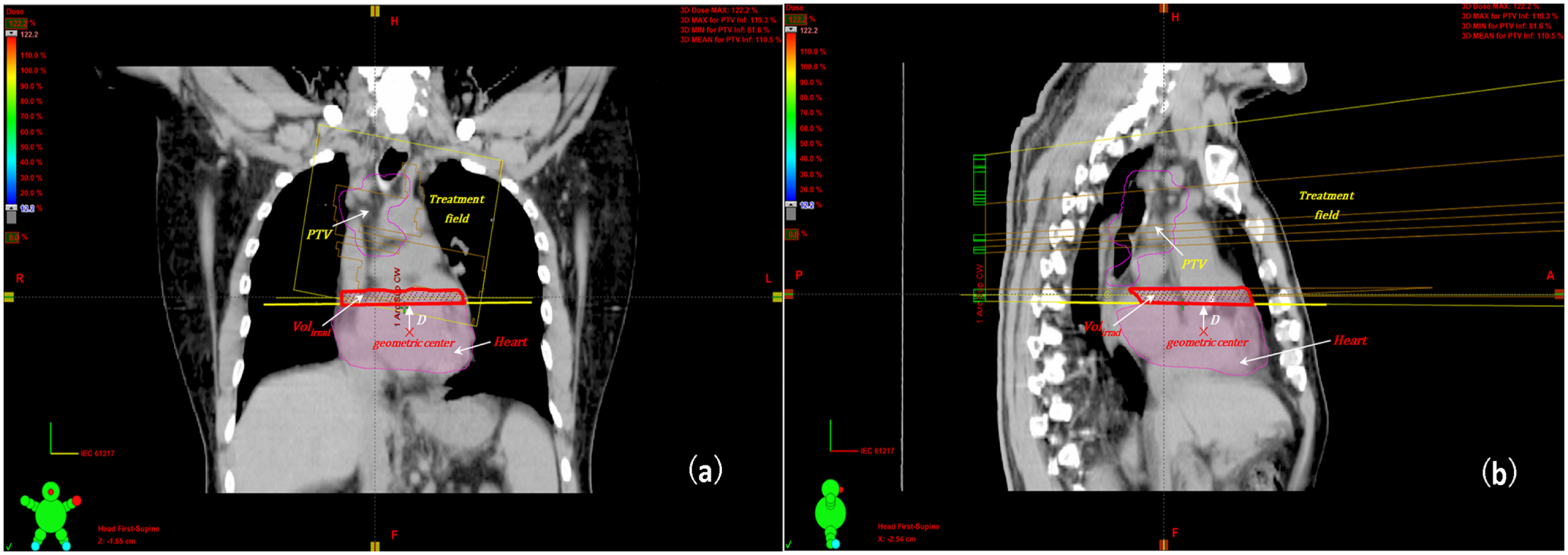
Definition of beam-to-heart distance and irradiated heart volume in lung cancer radiotherapy with a coronal view (a) and a sagittal view (b). The beam-to-heart distance (*D*) will be a positive value when the geometric center of the heart is located outside of the irradiated heart volume, and a negative one when it falls within the irradiated heart volume.

Long-term cardiac mortality has been shown directly correlated with mean dose to the heart [29–35], maximum heart dose [42–45], as well as V25 and V30 of the heart [25, 36]. Therefore, we have used mean and maximum heart doses, as well as V25 and V30 as dosimetric metrics to evaluate the cardiac exposure in the DCAT, IMRT and VMAT of lung cancer. The cardiac dose distribution were calculated with the full three-dimensional CT set. The dose-volume histograms (DVHs) for the whole heart were generated for each treatment plan. In addition, the effects of tumor laterality, the proximal distance between geometric center of the heart and the nearest edge of the treatment field (beam-to-heart distance, *D*) and irradiated heart volume (*Vol*_*irrad*_) on cardiac exposure were also investigated (Fig 1).

## Results

### Heart doses vs. tumor laterality

Among the 140 plans in this study, the mean dose to the heart ranged from 0 to 28.9 Gy, with an overall average of 4.5 Gy. Specifically, the mean doses to the heart were found to be 2.3, 5.2 and 4.6 Gy for the DCAT, IMRT and VMAT plan, respectively (Table 1). Four patients were delivered more than 20 Gy in MHD. There were 26 patients (18.6%) who received more than 105% of prescription dose to the heart, and the tumors were located at the lower lobe and of large volume.

**Table 1:** Comparison of mean and maximum doses to the heart.

	Average mean dose ± SD (Gy)	Average maximum dose ± SD (Gy)	Average V30 ± SD (%)	Average V25 ± SD (%)
**Treatment modalities**				
DCAT	2.3 ± 3.0	11.6 ± 15.7	0.1 ± 0.5	0.2 ± 1.1
IMRT	5.2 ± 7.0	26.7 ± 28.6	3.5 ± 8.7	4.5 ± 10.1
VMAT	4.6 ± 5.2	30.9 ± 27.5	2.9 ± 6.3	4.0 ± 8.1
**Tumor locations**				
LUL	1.9 ± 2.8	22.5 ± 30.5	2.0 ± 4.4	2.6 ± 5.6
LLL	6.8 ± 7.1	39.8 ± 24.8	11.3 ± 18.5	14.2 ± 21.1
RUL	1.0 ± 2.3	7.2 ± 16.8	1.4 ± 4.4	1.6 ± 5.1
RML	3.7 ± 4.1	21.3 ± 22.0	6.1 ± 13.3	8.9 ± 16.2
RLL	9.4 ± 7.2	38.9 ± 26.1	16.2 ± 19.2	19.8 ± 21.0

The average value over all assessed patients was calculated together with its standard deviation (SD).

There were 87 right-sided tumors (RUL, RML and RLL) and 53 left-sided ones (LUL and LLL) in this study. The mean doses to the heart were 4.1 Gy for the left tumors while 4.8 Gy for the right ones. As shown in Table 1, there were less differences between the left-sided and right-sided tumors than that reported in breast cancer [12–16]. In addition, the heart was generally better protected from irradiation beams when the tumors were located in the upper lobes (RUL and LUL) as compared to the middle and lower lobes (RML, RLL and LLL). The average mean dose to the heart for lower lobe tumors was 3.6–9.4 times more than that for upper lobe tumors.

In 55 patients (39.3%), the heart received scattered dose alone without direct irradiation by the treatment beams and the average scattered dose was 0.3 Gy. For the other 85 patients (60.7%), a part of the heart was in the radiation fields. Of these 85 patients, 31 had >50% of the heart volume in the fields, and 12 had >90% of the heart volume irradiated. All plans were evaluated to follow the RTOG recommendation such that 60 Gy to <1/3, 45 Gy to <2/3, and 40 Gy to <100% of the heart. In this work, V30 was < 46% for all the 140 plans suggested by Wei *et al*. [25], but V25 was found > 10% in 15 plans. Marks *et al*. suggested that V25 for the whole heart should be less than 10% to avoid long-term cardiac mortality [36].

Note that the standard deviations of the average dose values reported in Table 1 were large, as compared to the standard deviations of heart doses in breast cancer radiotherapy [11]. This is caused by the large variability in lung cancer radiotherapy in terms of tumor volume, tumor laterality, beam-to-heart distance, prescription dose, as well as treatment modality.

### Effect of beam-to-heart distance on cardiac exposure

**Fig 2:**
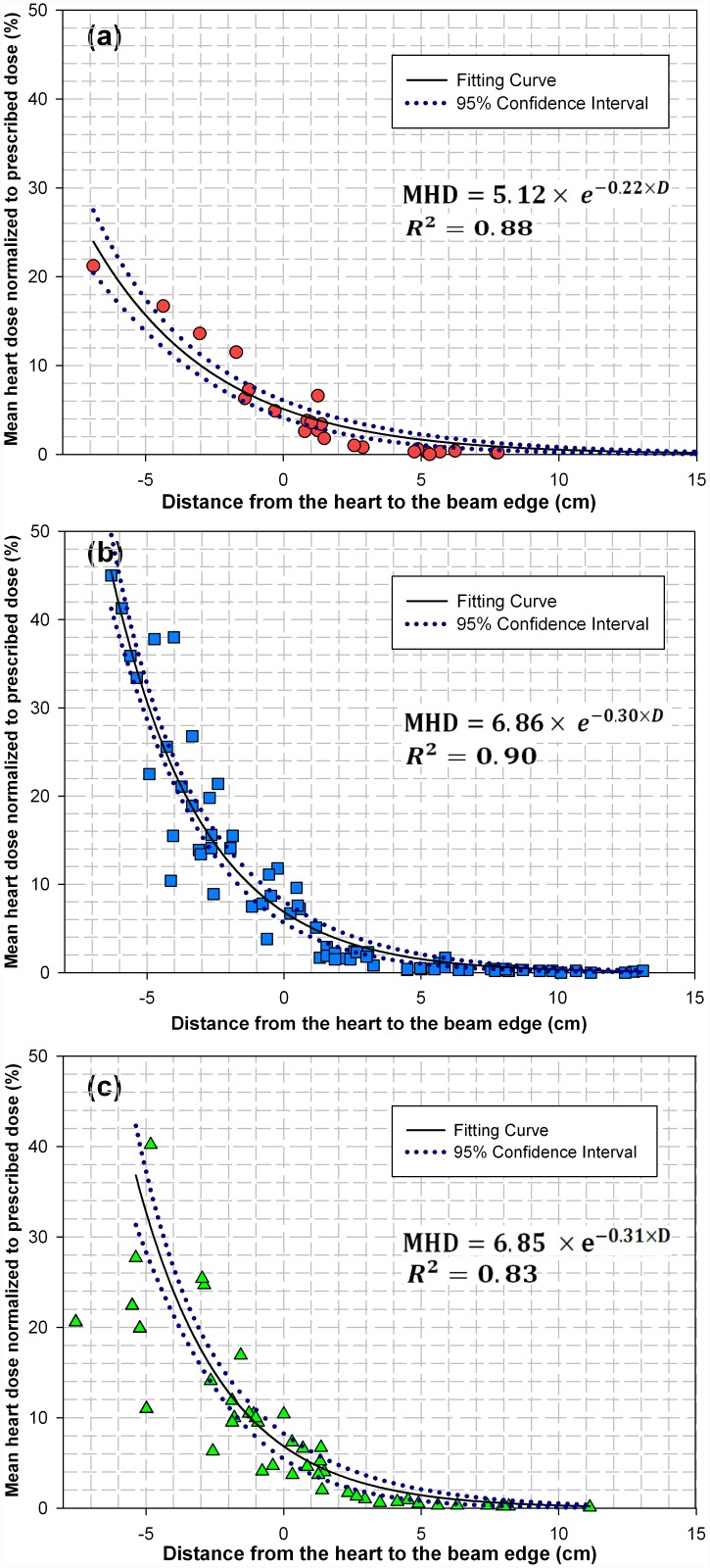
The mean dose to the heart as a function of distance from the heart to the beam edge for (a) DCAT, (b) IMRT and (c) VMAT plans.

Shown in Fig 2 were three scatter plots depicting the relationship between the mean heart dose (MHD) and the beam-to-heart distance (*D*) in the DCAT, IMRT and VMAT of lung cancer. As shown clearly, the radiation dose to the heart depends strongly on the beam-to-heart distance, irrespective of treatment modalities. The dependence followed an exponential decaying trend, as shown by the fitting curves which take a form of MHD = *a* × *exp*(-*b* × *D*). The fitting parameters (*a*, *b*) were found to be (5.12, 0.22) for the DCAT, (6.86, 0.30) for the IMRT, and (6.85, 0.31) for the VMAT plans, respectively. When D is larger than zero, the heart was delivered by the scattered dose from the radiation beams. The mean dose to the heart increased with the decrease of the beam-to-heart distance.

### Effect of irradiated heart volume on cardiac exposure

**Fig 3:**
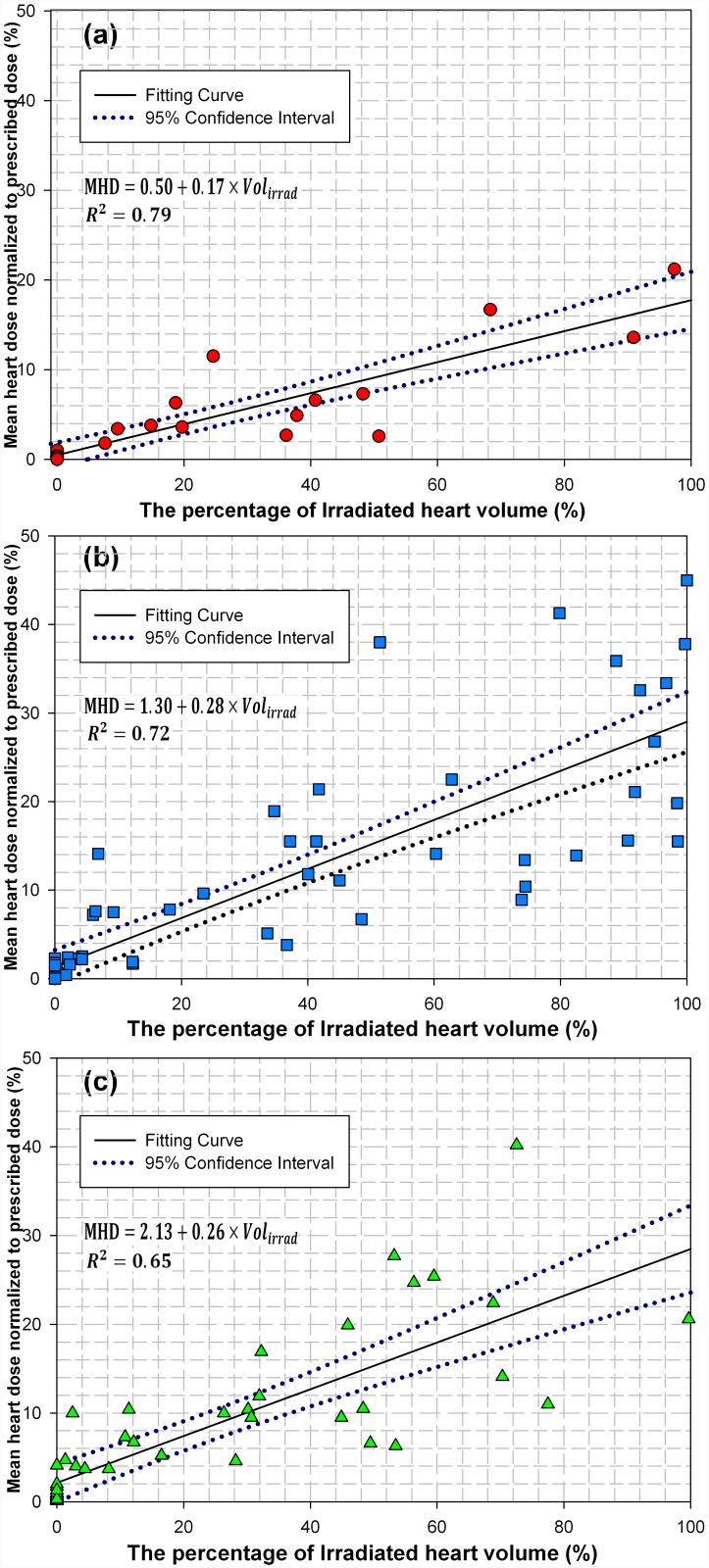
The mean dose to the heart as a function of irradiated heart volume for (a) DCAT, (b) IMRT and (c) VMAT plans.

Fig 3 compared the three treatment modalities in terms of cardiac exposure as a result of partial irradiation by the treatment beams. Generally speaking, the mean dose to the heart increased linearly with the irradiated heart volume (*Vol*_*irrad*_) for the three modalities, with the fitting functions taking a form of MHD = *c* + *d* × *Vol*_*irrad*_. The fitting parameters (*c*, *d*) were found to be (0.50, 0.17) for DCAT, (1.30, 0.28) for IMRT, and (2.13, 0.26) for VMAT plans, respectively. This means that the mean dose delivered to the heart increased by 1.7% for DCAT, 2.8% for IMRT, and 2.6% for VMAT for each increase of 10% heart volume irradiated by the radiation beam.

## Discussion

### Reduction in cardiac exposure by modern radiotherapy techniques since 1990s

Shown in Table 2 is the comparison of mean heart dose received from different radiotherapy regimens used in the past. In the radiotherapy of lung cancer, the mean heart dose determined in this study for all the 140 plans delivered from 2007 to 2014 was 4.5 Gy (range, 0–28.9), which is significantly lower than 24.7 Gy from 1995 to 2007 [40] and 15.5 Gy from 2003 to 2006 [26]. As pointed out by Demirci *et al*. [10], modern approaches usually led to lower heart doses in the radiotherapy of breast cancer as a result of better targeting and use of higher energy photon beams, in contrast to the old techniques where the relatively high doses per fraction, photon irradiation of the bilateral internal mammary nodes, and orthovoltage or Co-60 equipment had been used. In modern lung cancer radiotherapy, cardiac exposure was largely reduced with image-guided targeting and inverse planning-assisted normal tissue sparing.

**Table 2:** Comparison of mean heart dose in the radiation treatment.

Cancer	Years of diagnosis	Population	Country	Dose to the heart (Gy)		Ref.
				Left-sided	Right-sided	
**Lung Cancer**	1995–2007	250	Denmark	24.7*		[40]
**Lung Cancer**	2003–2006	24	USA	15.5		[26]
**Lung Cancer**	2007–2014	140	USA	4.1	4.8	Our study
**Breast Cancer**	1950s-1990s	-	-	0.9–14	0.4–6	[14]
**Breast Cancer**	1958–2001	2168	Sweden & Denmark	6.6	2.9	[13]
**Breast Cancer**	1976–2006	43802/28332	Denmark/ Sweden	6.3	2.7	[12]
**Breast Cancer**	1977–2001	681/130	Denmark/ Sweden	6/7.3	2-3/3.2	[15]
**Breast Cancer**	After 2005	48	USA	2.17	0.62	[16]
**Breast Cancer**	2006	55	UK	2.3	1.2–2	[11]
**Peptic ulcer disease**	1936–1965	3719	USA	1.6–3.9		[28]

*defined as mean dose level that exceeded the doses that 75% of the patients received.

In this study, we retrospectively investigated treatment plans for 140 lung cancer patients treated with DCAT, IMRT or VMAT. Clinically speaking, all the three modalities provided adequate PTV coverage and acceptable sparing of critical structures, but the dose to the heart in DCAT plans was the lowest among the three modalities (Table 1, Figs 2 and 3). One reason for the largest heart sparing in DCAT plans could be that patients with smaller tumor volumes were dominantly treated with DCAT at our institution [46]. For example, the average PTV in DCAT plans was 51.7 (range, 10.1–268.5) cm^3^, compared with 186.9 (range, 11.7–945.2) cm^3^ in IMRT plans and 231.0 (range, 14.3–1266.8) cm^3^ in VMAT plans.

### Cardiac toxicity in lung cancer radiotherapy

Several studies indicated that radiotherapy is associated with the incidence of RIHD for lung cancer patients [26, 37, 39–41]. Although the dose to the heart in lung cancer patients was much higher than that in breast cancer or peptic ulcer disease patients (Table 2), there has been no significant data yet to demonstrate the relationship between the heart dose and RIHD in lung cancer radiotherapy [26, 40], while the corresponding relationship has been extensively reported in breast cancer [13], pediatric cancer [23], and peptic ulcer disease [27]. Two main factors contributed to the lacking of data: the low survival rates and the long latency of radiation-induced heart disease for lung cancer patients. The 5-year relative survival rates for lung cancer patients were 17%, while the survival rate for breast cancer at a localized stage was as high as 99% [47]. The risk of cardiovascular disease began to increase 10 years after irradiation for breast cancer [7–9, 12, 34] and Hodgkin’s disease [19–21].

There were no records of patients who died of cardiac toxicity in our study. Notably, one patient with pre-existing coronary artery disease and congestive heart failure received a maximum heart dose of 67.1 Gy (112% of prescription dose) in his VMAT plan, and developed a pericardial effusion right after radiotherapy. Therefore, it is important to reduce cardiac exposure as much as possible for lung cancer patients, especially for those with pre-existing cardiac diseases [12, 13]. Considering the high dose level to the heart and correlation of RIHD with heart dose in the radiotherapy of other cancers, the dose to the heart could be a potential risk factor for RIHD in lung cancer radiotherapy. A database with longer follow-up on the radiation-related heart diseases in lung cancer radiotherapy would be highly desirable to carry out a systematic analysis of the long-term mortality of RIHD in the modern radiotherapy of lung cancer [32].

### Tumor laterality, beam-to-heart distance and irradiated heart volume

Some recent studies on breast cancer radiotherapy indicated an increased risk for cardiac dysfunction for the left-sided tumors [8, 9, 12, 13, 17]. Table 2 also showed that the mean dose to the heart was higher for the left-sided tumors than the right-sided ones. In this work, however, the mean heart dose was found to be 4.1 Gy for the left-side tumors while 4.8 Gy for the right-sided ones. The lateral difference of cardiac exposure was much less in lung cancer radiotherapy as compared to breast cancer radiotherapy. This is partially due to the proximity of the heart to the tangential field borders when left breast tumor was treated. Another reason is that in the DCAT, IMRT and VMAT treatments of lung cancers, the radiation beams were optimally set to conform the dose distribution to the tumor volume with beams coming from all solid angles, depending on the priorities set in different modalities to avoid the excessive dose to the heart and other OARs. As such, the lateral difference of cardiac exposure in our cohort of 140 lung cancer patients was much less as compared to breast cancer radiotherapy, with 4.1 Gy for the left-side tumors while 4.8 Gy for the right-sided ones. Further study with more lung cancer cases would be needed to confirm this clinical observation in the future.

The mean heart dose was found to decrease exponentially as the beam-to-heart distance increased (Fig 2). As such, the beam-to-heart distance can be used to estimate the mean heart dose and subsequent radiation-induced heart disease in different treatment modalities for lung cancer radiotherapy [33], similar to the maximum heart distance used for mean heart dose prediction in breast cancer radiotherapy [18].

As shown in Fig 3, the mean heart dose increased linearly with the irradiated heart volume, regardless of the treatment modalities used for lung cancer radiotherapy. Specifically, for every 10% increase in irradiated heart volume, the percentage mean heart dose increased by 2.8% for the IMRT plans, 2.6% for the VMAT plans and 1.7% for the DCAT plans. Given that the dose distributions were calculated by treatment planning system to within ±2% for dose uncertainty, and dose assessed from different institutions at different times varied largely, it is therefore very beneficial to consider the beam-to-heart distance and the irradiated heart volume collectively in the radiotherapeutic management of lung cancer in order to maximize the heart sparing [17]. Furthermore, these indexes can be used to predict the risk for RIHD if the integrated system of cardiac toxicity in lung cancer radiotherapy is established in the future studies.

This study serves as the first step toward gaining a deeper understanding of the cardiac exposure during the radiotherapy of lung cancer in the modern era. The cardiac exposure was reduced by the modern techniques for lung cancer patients, but it was still higher than that among other cancer patients. We provided two indexes (i.e., beam-to-heart distance and irradiated heart volume) to assess the mean heart dose which could be used as the potential risk factors associated with RIHD instead of using only the mean heart dose in the future study.

This study assessed the dose to the whole heart without dose information of sub-volume and structures in the heart such as coronary artery and pericardium. Although 140 plans delivered with DCAT, IMRT or VMAT between 2007 and 2014 have been investigated, no correlation study of RIHD with the cardiac exposure has been completed as a longer follow-up would be required to determine the pattern and prevalence of radiation-induced cardiac diseases in the lung patients.

## Conclusion

The mean dose to the heart in 140 lung cancer patients treated with DCAT, IMRT or VMAT was 4.5 Gy, lower by 82% compared with the conventional techniques used in the 1990s. The lateral difference of the mean heart dose was small, with 4.1 Gy for the left-sided tumors vs. 4.8 Gy for the right-sided ones. In addition, the mean heart dose was found to decrease exponentially with the beam-to-heart distance while increasing linearly with the irradiated heart volume, which could be used to investigate the correlation of RIHD risk with heart exposure in lung cancer radiotherapy.

## Supporting Information

S1 Table The mean dose to the heart as a function of distance from the heart to the beam edge for (a) DCAT, (b) IMRT and (c) VMAT plans. These were the original data for Fig 2.
(XLSX)

S2 Table The mean dose to the heart as a function of irradiated heart volume for (a) DCAT, (b) IMRT and (c) VMAT plans. These were the original data for Fig 3.
(XLSX)
